# The Real Cytotoxic Effect of Artemisinins on Colon Cancer Cells in a Physiological Cell Culture Setting. How Composition of the Culture Medium Biases Experimental Findings

**DOI:** 10.3390/ph14100976

**Published:** 2021-09-26

**Authors:** Dagmara Otto-Ślusarczyk, Magdalena Mielczarek-Puta, Wojciech Graboń

**Affiliations:** Chair and Department of Biochemistry, Faculty of Medicine, Medical University of Warsaw, Banacha 1, 02-097 Warsaw, Poland; dotto@wum.edu.pl (D.O.-Ś.); wgrabon@wum.edu.pl (W.G.)

**Keywords:** artemisinin, dihydroartemisinin, ferroptosis, interleukin-6, linoleic acid, holotransferrin, colorectal cancer

## Abstract

Artemisinin (ART) and dihydroartemisinin (DHA) are anti-malaria drugs but also exhibit huge anticancer potential based on ferroptosis driven by iron-dependent lipid peroxidation. This study was conducted on primary (SW480), metastatic (SW620) colon cancer, and noncancerous HaCaT cells at pharmacologically relevant drug concentrations (1–8 µM) and in the presence of holotransferrin (TRFi 50 µM) and linoleic acid (LA 20, 40 µM) at physiological levels. ART and DHA showed the growth inhibitory potency which was significantly increased in the presence of LA or/and TRFi. The IC_50_ for ART or DHA, LA40 and TRFi combination in both cancer cell lines ranged 0.14–0.69 µM whereas no cytotoxic effect was observed for HaCaT cells (SI = 202–480). Almost all experimental settings revealed late apoptosis in both cancer cell lines, but not in normal cells. The percentage of late apoptotic cells increased with LA concentrations and was intensified after TRFi addition. The strongest pro-apoptic effect was exhibited by ART or DHA, LA40, and TRFi combination. More interestingly, we found a stimulatory effect of TRFi on IL-6 synthesis. The present study using LA and TRFi which are inherent blood components revealed high antitumor artemisinin activity in concentrations achievable after drug administration to cancer patients without toxic effects on normal cells.

## 1. Introduction

Colorectal cancer (CRC) is the third-most-common malignant tumor in Poland and the fourth-rated as the leading cause of deaths in worldwide. Although, early screening, diagnosis improvement, and CRC treatment increased the 5-year survival rate of patients, to some extent, the proliferation and metastasis of tumor still present challenges in the treatment of CRC [1]. Although various treatment options are available (surgery, radiotherapy, and adjuvant chemotherapy), in many cases these therapies are marked by a high level of toxicity to healthy cells, and drug resistance quickly develops in some treatment regimens [2,3,4]. One major challenge to reducing the adverse effects of the cancer burden is to develop highly effective drugs with specificity on cancers but little or no side effects on normal cells.

Recent studies have demonstrated that compounds present in traditional medicinal plants such as artemisinin and its derivatives possess therapeutic potential for CRC [5].

**Figure 1:**
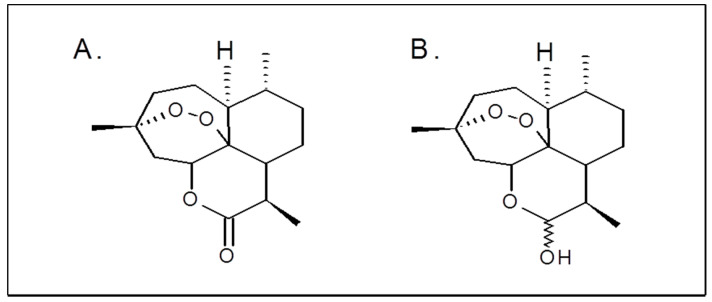
Chemical structures of artemisinin (**A**) and dihydroartemisinin (**B**)**.**

Artemisinin (ART), an organic compound isolated from the plant *Artemisia annua.* Artemisinin derivatives such as artesunate, artemether, and their metabolite dihydroartemisinin (DHA) are widely used in clinical practice as antimalarial drugs (Figure 1A,B) [6,7].

In addition, preclinical studies in vitro and in vivo have shown that all mentioned compounds exhibit a strong antitumor activity against various human cancer cells, including colorectal cancer [8,9,10]. 

Research on ARTs has shown that its antitumor mechanism is similar to its antimalarial action and is based on iron activation [11]. In consequence, ART and DHA may sensitize cancer cells to ferroptosis—a process dependent on the intracellular iron presence [12,13]. ARTs contain a unique endoperoxide moiety that can react with cellular iron to form cytotoxic-free radicals causing peroxidation of cell membrane lipids [14,15,16]. It is known that cancer cells are characterized by abnormally high iron levels promoting reactive oxygen species (ROS) generation which results in oxidative cellular damage [17,18,19]. In comparison to normal cells, cancer cells also have more transferrin receptors on the cell surface in order to accumulate the iron necessary for their proliferation. ARTs, by reacting with iron in the malignant cells destroy them, but normal cells unsaturated with iron remain virtually unaffected by these agents [20,21,22]. Thus ARTs may provide targeted anti-cancer therapy, making the tumor cells selectively more susceptible to the cytotoxic effects [23].

Moreover ARTs potentiate ferroptosis by stimulation of ferritinophagy—the process of autophagic ferritin degradation resulting in increased free iron levels. This is followed by further increase in ROS level via the Fenton reaction [24,25]. 

ROS delivered by ART activity and the Fenton reaction induce lipid peroxidation, disintegration of lipid membranes, and finally, ferroptotic cancer cell death. Linoleic acid (LA) and especially its endogenous product arachidonic acid (AA) are esterified to PUFA-containing membrane phospholipids. They are especially vulnerable to oxidation by ROS formed in the result of iron–ART interaction [26,27]. In turn, unsaturated fatty acid composition affects the generation of free radicals [28]. Thus the amount of PUFAs is a key factor for the intensity of lipid peroxidation that can occur in cells. As yet the majority of studies on anti-tumor ferroptotic ARTs effects in vitro have been carried out without LA in culture media. It results in PUFA-devoid membrane lipid composition not occurring in tumor cells in vivo. In our opinion, results of these studies are unreliable since lack of LA in media prevents intracellular synthesis of AA—a crucial components of phospholipids undergoing iron-dependent peroxidation leading to ferroptosis. 

Obviously, an iron-binding transferrin should also be an essential component of culture media in studies on ferroptosis triggered by ARTs.

Most experimental studies have been carried out at high ART, DHA, and LA concentrations never achieved in human plasma [29,30]. For our experiments we have chosen ART and DHA concentrations which correspond with these occurring in plasma (1 or 2 µM) and the tumor environment (8 µM). The latter concentration was selected based on observations of drugs accumulation in cancer tissue [31].

Accordingly, in our study for the first time we investigated the antitumor effect of ARTs in pharmacologically achievable concentrations with LA and TRFi present in the culture medium at concentrations found in human serum. 

## 2. Results

### 2.1. Cytotoxic Activity

To establish the cytotoxic effects of ART and DHA, they were of screened for their in vitro antitumor activity on a human cancer cell lines (SW480 and SW620) versus the control (HaCaT) cell line. All tested cells were cultured for 72 h in four different settings: with ART or DHA alone (1, 2, and 8 µM), with ART and LA (20 and 40 µM), or TRFi (50 µM) addition and with ART and both LA and TRFi. In order to determine the half maximal inhibitory concentration (IC_50_) some studied settings were also performed with non-physiological ART and DHA concentrations (50–250 µM). Moreover, a cytotoxic potency against tumor cells for tested compounds was expressed as a selectivity factor (selectivity index, SI).

All studied settings (ART, DHA, or mixtures) were more cytotoxic for cancer than for control HaCaT cells. The IC_50_ values calculated for HaCaT cells were several times higher compared to the studied colon cancer (SW480, SW620) cells, while the mixture of ART or DHA and LA with TRFi showed about an eight-fold higher cytotoxicity activity against cancer cells in comparison to the same mixture without TRFi (Table 1).

The highest growth inhibitory potency was denoted for settings ART-LA 40 TRFi (IC_50_ = 0.69 ± 0.01 µM and IC_50_= 0.6 ± 0.04 µM) and DHA-LA 40 TRFi (IC_50_ = 0.24 ± 0.02 µM and IC_50_ = 0.14 ± 0.01 µM) for SW480 and SW620 cells, respectively, and also DHA-LA 20 TRFi (IC_50_ = 0.38 ± 0.09 µM) only in SW480 cells.

Furthermore, the highest selectivity index (SI) was also achieved for ART-LA 40 TRFi (SI = 202 for SW480 and 225 for SW620), and DHA-LA 40 TRFi (SI = 288 for SW480 and 480 for SW620), and also DHA LA 20 TRFi (SI = 273 for SW480).

### 2.2. The Effect of Artemisinin and Dihydroartemisinin on Viability of Human Cancer and HaCaT Cells

**Figure 2:**
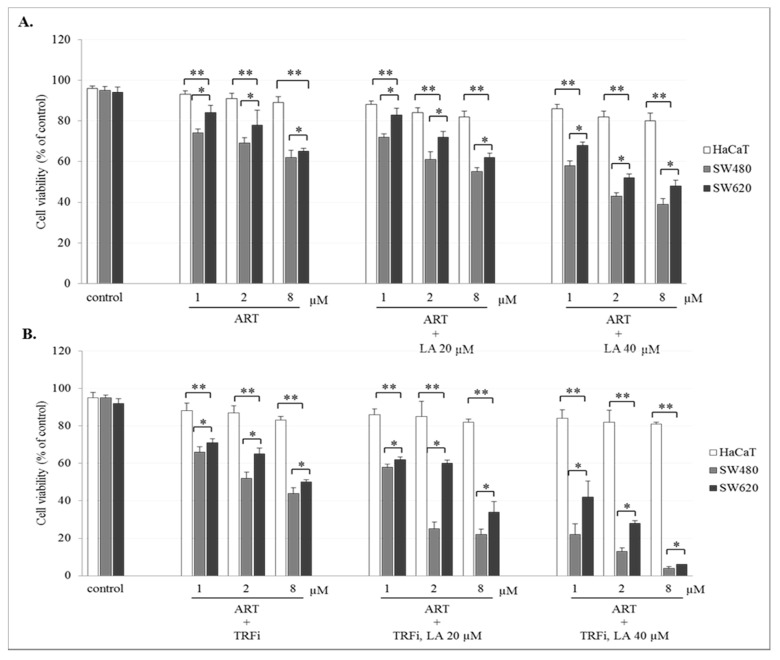
Effect of ART alone and ART with LA addition (**A**), ART-TRFi alone and ART-TRFi with LA addition (**B**) on viability of SW480, SW620, and HaCaT cells. Cells were treated for 72 h with various concentrations of tested compounds ART (1, 2, and 8 μM), LA (20 and 40 μM) and TRFi (50 μM). Cells viability was assessed by TB assay. Data are expressed as means ± SD. ** *p* < 0.001 as compared to control, * *p* < 0.001 as compared to HaCaT.

**Figure 3:**
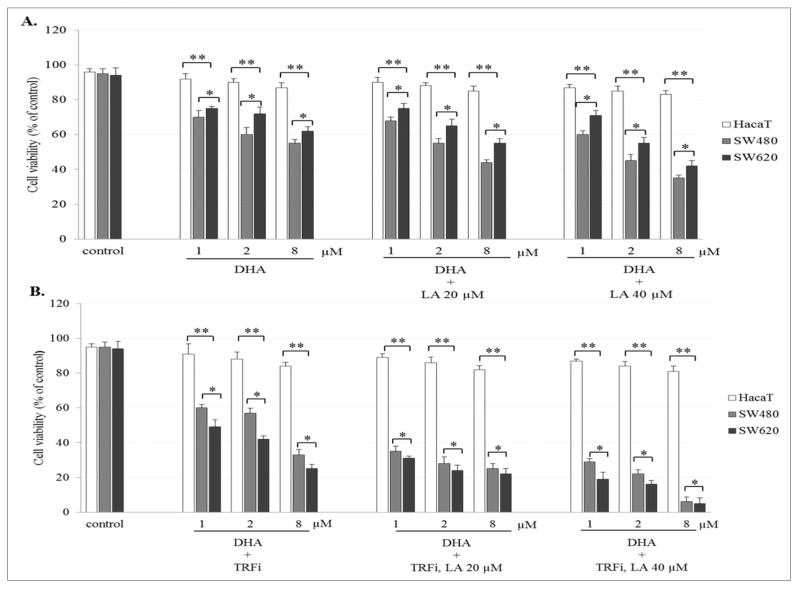
Effect of DHA alone, DHA with LA addition (**A**), DHA–TRFi alone and DHA-TRFi with LA addition (**B**) on viability of SW480, SW620, and HaCaT cells. Cells were treated for 72 h with various concentrations of tested compounds ART (1, 2, and 8 μM), LA (20 and 40 μM) and TRFi (50 μM). Cells viability was assessed by TB assay. Data are expressed as means ± SD. ** *p* < 0.001 as compared to control, * *p* < 0.001 as compared to HaCaT.

The incubation of SW480 and SW620 cells with ART or DHA alone or with LA addition showed a concentration-dependent effect on cell viability. The number of viable cancer cells in the presence of studied compounds was significantly lower (*p* < 0.001) in comparison to control (untreated cancer cells). The viability of cancer cells after ART (8 µM) or DHA(8 µM) with LA (40 µM) treatment was for 39 ± 2.8% and 35 ± 1.8% in SW480, and 48 ± 2.8% and 42 ± 2.1% in SW620 cells, respectively (Figure 2A and Figure 3A). After TRFi addition the number of living cancer cells was significantly lower (*p* < 0.001) in all studied cases in comparison to the cancer cells treated with ART and LA without TRFi and control cancer cells. In the presence of ART or DHA (8 µM), LA (40 µM) and TRFi cancer cells viability ranged from 4% to 6% (Figure 2B and Figure 3B). In contrast, in almost all studied settings the viability of HaCaT cells was significantly higher (*p* < 0.001) than that of SW480 and SW620 cells and ranged between 80% and 93%.

### 2.3. Apoptotic Activity of Artemisinin and Dihydroartemisinin

**Figure 4:**
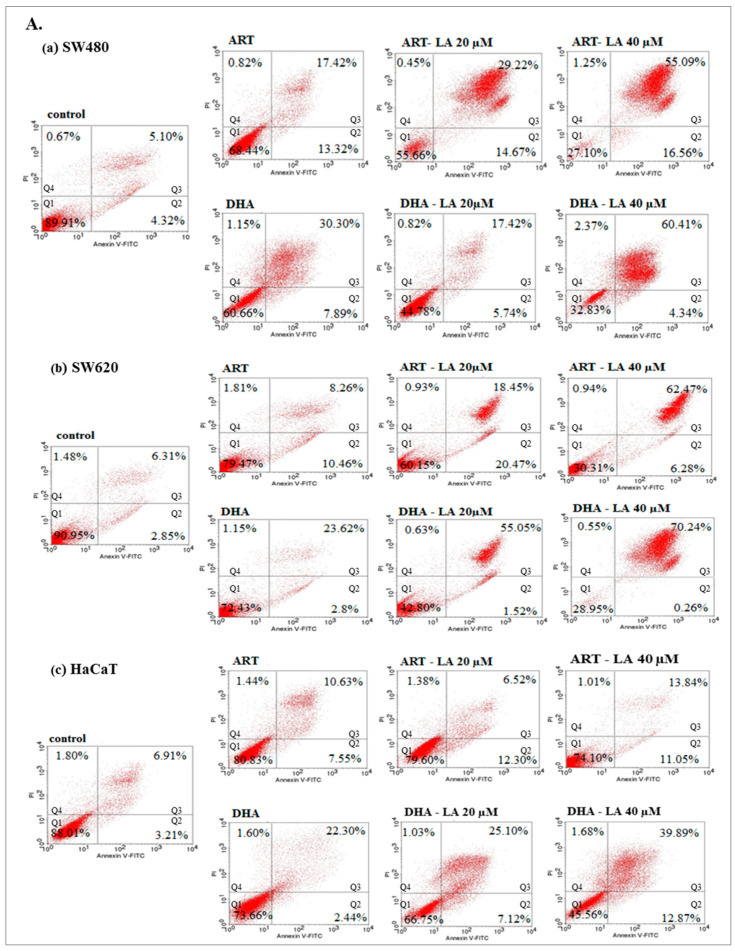

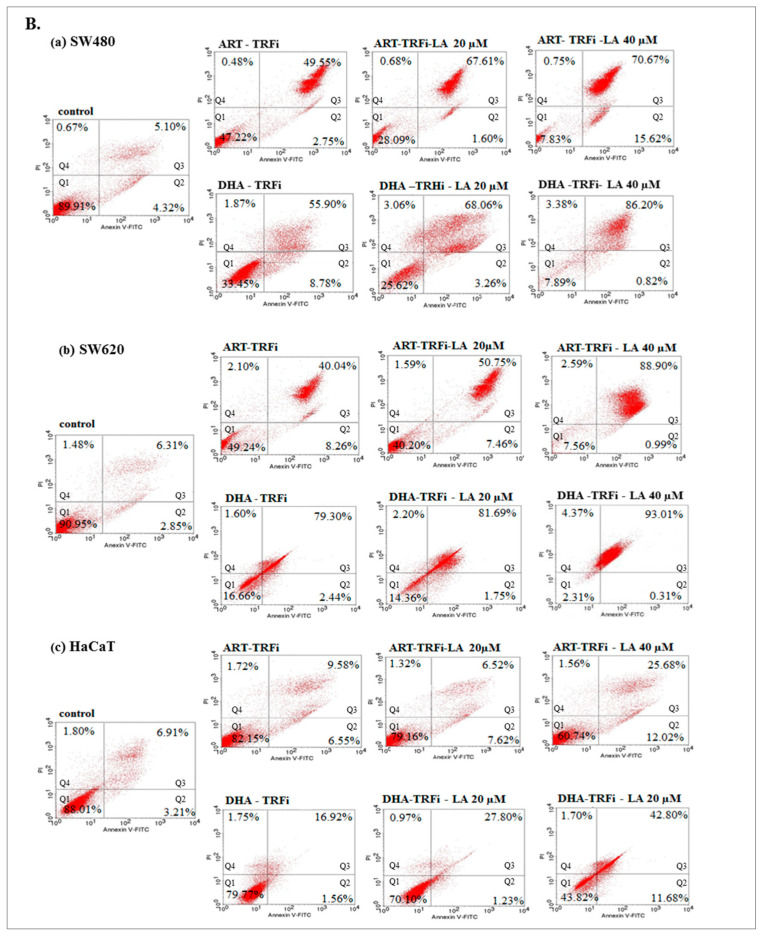
The effect of ART, DHA, and LA (**A**), ART and DHA with TRFi and LA (**B**) on late apoptosis in SW480 (**a**), SW620 (**b**), and HaCaT (**c**) cells detected with Annexin V-FITC/PI by flow cytometry. Diagrams show representative experiments. A total of 10,000 events were collected. The lower left quadrant (Q1) represent viable cells and the lower right quadrant (Q2) early apoptotic cells. The upper right quadrant (Q3) contains late stage apoptotic cells, and the upper left quadrant (Q4) necrotic cells.

To assess in vitro the anticancer mechanism of action, the effect of ART or DHA (2 µM) with/without LA (20, 40 µM) and TRFi on early and late apoptosis was provided by flow cytometry analysis (Figure 4A).

The incubation of SW480 and SW620 cells with tested compounds showed a significantly higher percentage of cells in late apoptosis, as compared to the controls (untreated cancer cells and HaCaT cells).

Except from ART and ART-LA 20 in SW620 cells the rest of the tested settings induced late apoptosis in cancer cell lines that ranged from 20% to 86% for SW480 and 18.45–93% for SW620 cells. The percentage of late apoptotic cells increased with LA concentrations and was intensified after TRFi addition. Incubation of SW480 and SW620 cells with ART or DHA, LA 40, and TRFi addition showed 90% of cells in late apoptosis. In contrast, the same settings in HaCaT cells induced lower rates of late apoptosis (25.68% for ART and 42.80% for DHA) (Figure 4B).

### 2.4. IL-6 Level Studies

**Figure 5:**
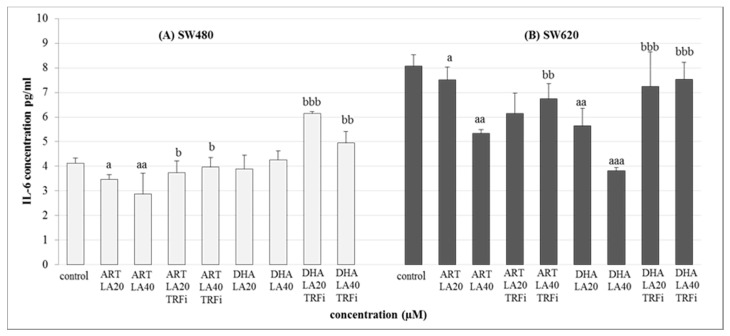
The effect of ART, DHA, and LA with or without TRFi on IL-6 levels in (**A**) SW480 and (**B**) SW620 cancer cells. Cells were incubated for 72 h with studied compounds at their IC_50_ concentrations. IL-6 levels in culture medium were measured by ELISA test. Data are expressed as means ± S.D. from three independent experiments performed in triplicate. **^aaa^** *p* < 0.001, **^aa^** *p* < 0.01, **^a^** *p* < 0.05 as compared to the control and **^bbb^** *p* < 0.001, **^bb^** *p* < 0.01, **^b^** *p* < 0.05 as compared to the ART or DHA with LA 20 or 40.

Both human cancer cell lines were treated with the IC_50_ concentrations of the most promising tested settings mixtures i.e., ART-LA (20 and 40 µM) and DHA-LA (20 and 40 µM), with/or without TRFi (Figure 5).

The significant decrease of IL-6 secretion was observed for both studied cancer cell lines in the presence of ART-LA (20 and 40 µM) without TRFi as compared to control (LA 20 µM) (*p* < 0.05, *p* < 0.01). The mixture of DHA–LA (20 and 40 µM) without TRFi significantly reduced IL-6 release only in SW620 cells (*p* <0.01, *p* < 0,001). Looking closer at the results, the strongest effect was identified for DHA and LA 40 µM. Interestingly, the addition of TRFi increased IL-6 concentration in all studied settings except ART-LA20 in SW620 cells (Figure 5).

## 3. Discussion

Colorectal cancer (CRC) causes significant morbidity and mortality worldwide. CRC rates are rising alarmingly in young adults, which may be due to lifestyle and genetic factors [32]. Modern anticancer therapies show better response and survival rates, but side effects and poor quality of life often lead to the treatment discontinuation, dose reduction, and manifestations of drug resistance. Therefore, new therapeutic measures are urgently needed to improve outcomes in the growing number of people diagnosed with CRC.

Anticancer properties of ART and its derivatives have been known and studied for many years. Data from many studies have revealed that artemisinin and its derivatives exhibit selective cytotoxicity against many types of cancer both in vitro and in vivo without inducing any toxicity in normal cells [33].

It should be noted that in our discussion we do not address the studies on artesunate, the other artemisinin derivative, because this compound is a prodrug transformed in the human body to DHA. Thus, artesunate studies in vitro cannot be translated to in vivo conditions because of a very low and short-time plasma prodrug concentration [10,30].

In the present work we found that IC_50_ of ART and DHA was 39 µM and 11.4 µM in SW480 cells and 42.8 µM and 11.9 µM in SW620 cells, respectively. This order of magnitude for IC_50_ was confirmed by Wang et al. (2018) who observed the increase of antitumor DHA activity on HCT-116 colon cancer cell line in dose- and time-dependent manner [34]. Kumari et al. (2017) found that there was no significant reduction in a viability of breast cancer cell lines with short-term ART treatment (12 h). After 24 h the viability of all cancer cells decreased in a dose-dependent manner but still in supra-pharmacological concentrations (32.14–88.08 μM) [35]. In turn, Efferth et al. (2004) revealed that IC_50_ value of ART in human leukemia cells and astrocytoma cells ranged from 3.3 µM to 11.5 µM after 7 days of treatment. These above data could also be evidence that the cytotoxic effect of ART develops over time [18].

Furthermore, we showed that treatment of cancer cells with ART and DHA alone in pharmacological concentrations (1–8 µM) only slightly decreased cancer cell viability (70–80%) in comparison to untreated cancer cells as well as HaCaT cells. It should be noted that the strong ART or DHA cytotoxic effect (10–60% of viability) revealed by other authors during short-term treatment (24–72 h) was obtained at drug concentrations never achievable in human serum (20–100 μM). According to Lu et al. (2018) the treatment of SW948 cells with 30 and 50 µM DHA for 48 h shown decreased their viability to 45% and 24% after 48 h [36]. In turn, Lu et al. (2014) showed 40% HCT-116 cell viability after treatment with 40 μM DHA for 72 h [37].

The above results would suggest a lack of efficacy of the antitumor effect of ART in vivo due to the failure to achieve effective serum concentrations, however efficacy has been obtained in experiments carried out without LA in culture medium.

LA and its intracellular product AA are necessary for effective ferroptosis as the susceptible target for peroxidation by ROS generated due to iron–ART interaction. It is known that key ferroptotic enzyme Acyl-CoA synthetase long chain family member 4 (ACSL4) uses AA to generate arachidonoyl-CoA (AACoA) as the substrate for membrane phospholipid synthesis [38,39].

Therefore, results obtained from experiments carried out in standard cultures without PUFA may indicate a ferroptosis-resistant state of cells containing only saturated and monosaturated fatty acids in membrane phospholipids [40].

LA (18:2, n-6) is not synthesized in the human body and has to be delivered with food. The free LA level in the plasma (20–30 µM) is maintained by the regular LA supply in the diet [41] and can be increased to 40–60 μM by supplementation [42]. In our previous studies we showed that physiological LA concentration (20 µM) did not affect growth and viability of SW480 and SW620 cells as well as HaCaT cells, whereas LA in concentrations achievable in blood serum after supplementation reduced viability of only cancer cells in concentration-dependent manner [43]. Thus, in the present study the anticancer effect of ART or DHA was assessed using LA in two concentrations—physiological (20 µM) and achievable in plasma by supplementation (40 µM).

We observed a significant decrease in IC_50_ for ART and DHA in both cancer cell lines at the physiological plasma LA level. This corresponds with the antitumor effect of the drugs administered to the body. This effect was enhanced in the presence of supplemental LA. Contrary to cancer cells, HaCaT cells were one order of magnitude less sensitive to ART and DHA cytotoxic effects. This indicates that normal body cells are not susceptible to both drugs (Table 1). Analogous findings were obtained for TB assay (Figure 2A and Figure 3A). To the best of our knowledge, there are no available studies in literature to compare our results.

TRFi present in the culture medium is necessary to determine ferroptotic activity of ART and DHA. To mimic the tumor environment we added TRFi at physiological concentration (50 µM). We found a decrease in IC_50_ for both ART and DHA at the presence of TRFi compared to drugs alone (4.58 vs. 39 and 1.64 vs. 11.4 in SW480 and 16.3 vs. 42.8 and 10.3 vs. 11.9 in SW620 for ART and DHA, respectively), which was more pronounced in primary colon cancer cells. Consequently, HaCaT cells were more resistant to combined ART or DHA and TRFi cytotoxic effects compared to drugs alone than cancer cells (203 vs. 187.1 for ART and 116 vs. 129.2 for DHA, respectively) (Table 1). Similar results were obtained for TB assay (Figure 2B and Figure 3B) The present data strongly suggest the need for research on ferroptotic artemisinin activity using culture media supplemented with TRFi. Our findings are consistent with shown by other authors [44,45]. According to Deng et al. (2013) TRFi significantly enhanced the growth suppression induced by artemisinin (50–150 µM) against SMMC-7721 cells within 72 h, which indicates that TRFi could sensitize the anti-growth effect elicited by ART [44]. In turn, Zhang et al. (2015) found that drug carriers with transferrin (Tf) and ART (HA-MWCNTs/Tf@ART) had higher inhibition efficiency on MCF-7 (human breast cancer) cells than drug carriers with ART (HA-MWCNTs@ART) or ART alone (1–50 µM) during 24–72 h incubation. The authors suggest that the special mechanism of action for ART and Tf meant that this drug delivery system greatly enhanced the pharmacological activity at the targeted cells [45]. Xie et al. (2010) showed that the complex of holo-transferrin-tagged DHA (DBAH-TH) was about 170 times more potent than DHA in killing MCF-7 cells and almost 300 times more potent in HNB cells (human normal breast) [46].

Finally, we examined the antiproliferative effect of ART and DHA on cancer and control cells cultured in medium containing both LA and TRFi in physiologically achievable concentrations. This resulted in further decrease in IC_50_ for both cancer cell lines which corresponded with drugs concentrations attainable in plasma. Cytotoxic ART and DHA activities in cancer cells are comparable with IC_50_ of doxorubicin—the standard cytostatic drug. However, ART or DHA treatment is superior to DOX therapy due to much lower cytotoxicity on normal cells (SI 202 and 288 vs. 0.38 in SW480 and 225 and 480 vs. 1.11 in SW620 for ART and DHA, respectively). This allows for continuous administration without adverse effects associated with DOX management. Moreover, ART or DHA oral therapy is more convenient compared to intravenous DOX administration.

In the present study we found that ART and DHA in pharmacological concentration (2 µM) induced a higher percentage of late apoptosis in cancer cells than HaCaT cells after 72 h of incubation. Lu et al. (2014) [37] shown early apoptosis at 10, 20, and 40 μM DHA concentrations in HCT-116 cells after 24 h, whereas Jia et al. (2016) obtained early apoptotic gallbladder cancer cells after treatment with ART (20 µM) within 24 h [47]. Our data revealed that the presence of LA enhanced the apoptic effect of both studied compounds. This effect was dose-dependent for LA and additionally intensified by TRFi addition in contrast to HaCaT cells. Very extensive late apoptosis after 72 h indicates rapid and effective cytotoxic activity of ART and DHA against colon cancer cells. Studies obtained by Kim et al. (2014) showed higher percentages of early and late apoptosis after 24 h incubation in glioblastoma cells treated with DHA in combination with TRFi as compared to DHA alone [48]. According to Zhang et al. (2015) more early stage apoptotic MCF-7 cancer cells were observed after treatment with HA-MWCNTs/Tf@ART in comparison to ART during 24 h of incubation [45]. The higher percentage of early apoptosis observed after short-time incubation (24 h) may indicate that the mechanism of artemisinin action is time-dependent. Additionally, more cells in the stage of late apoptosis observed in present work may result from not only TRFi but also linoleic acid. Our previous findings showed that LA alone at physiological concentration (20 µM) had low pro-apoptic effect, comparable with control (no LA in medium) [35]. However, the presence of LA at above concentration potentiates the apoptic effect of ART or DHA alone. It confirms that this effect is underestimated using the standard cell culture media without LA.

Multifunctional pro-inflammatory cytokine IL-6 was found in multiple cancer cell lines and tissues of cancer patients [49] what suggests that this protein is involved in the pathogenesis of various cancers [50,51]. Moreover, its elevated expression has been related to advanced stage of the disease and lower survival of patients with CRC [52]. There are many papers concerning the influence of IL-6 on systemic and tumor iron level but not iron on IL-6 synthesis. It known that IL-6 decreases transferrin (negative acute phase protein) level. Therefore in the present work we evaluated an effect of transferrin—a necessary ferroptotic factor on IL-6 synthesis and release by colon cancer cells. Our findings clearly show stimulation of IL-6 release from colon cancer cells by TRFi which indicates mutual IL-6 TRFi level regulation by tumor cells. This would suggest a negative effect of IL-6 on iron availability for cancerous cells giving protection against ferroptosis. However, it was found that SW480 cells are able to accumulate iron through increased expression of iron import proteins including transferrin receptor 1 (TfR1) and decreased expression of iron export proteins [53]. In this context our results seem to be very promising indicating that maintained iron uptake and accumulation by colon cancer cells promote ferroptotic artemisinin toxicity. This mechanism might be explained by the Latin sentence: “Qui gladio ferit, gladio perit”, which translates as “Who lives by the (iron) sword dies by the (iron) sword”.

## 4. Materials and Methods

### 4.1. Chemicals and Reagents

Artemisinin (ART), dihydroartemisinin (DHA), linoleic acid (LA), holotransferrin (TRFi) and thiazolyl blue tetrazolium bromide were purchased from Sigma-Aldrich (St Louis, MO, USA). Fetal bovine serum (FBS), PBS, 0.25% trypsin-0.02% EDTA, and penicillin/streptomycin were supplied by Gibco BRL (San Francisco, CA, USA). High glucose medium DMEM, MEM with Earle’s balanced salt solution (EBSS), and 1 M HEPES were obtained from Thermo Scientific (Waltham, MA, USA). FITC Annexin V Apoptosis Detection Kit I and all cell culture plastics were purchased from Falcon, Becton-Dickinson (Franklin Lakes, NJ, USA).

### 4.2. Drug Preparation

Artemisinin, dihydroartemisinin, and linoleic acid were dissolved in ethanol (99%) to obtain a 10 mM stocks solution for ART, DHA, and 90 mM for LA. Holo-transferrin was dissolved in in Milli-Q water to obtain 10 mM stock solution. All compounds were stored at 4 °C and 1 mM or 0.1 mM stock solutions were prepared in pure fresh recommended medium. Final dilutions of 1, 2, and 8 µM of ART and DHA and of 20 and 40 µM of LA and 50 µM of TRFi were used in the treatment of cells. Fresh concentration stock solutions were used for each experiment.

### 4.3. Cell Culture and Treatment

The human cell lines SW480 (primary colon cancer), SW620 (lymph node metastatic from the same patient as SW480), and HaCaT (immortalized keratinocyte) were obtained from the American Type Culture Collection (ATCC, Rockville, USA). SW480 and SW620 cells were grown in MEM (minimal essential medium, ThermoSci, USA), and HaCaT in DMEM high glucose (Dulbecco’s Modified Eagle’s Medium, Biowest SAS, France) supplemented with 10% FBS (fetal bovine serum), HEPES (20 mM), and antibiotics (100 U/mL of penicillin and 100 μg/mL of streptomycin). The cells were maintained in a humidified incubator at 37 °C/5% CO_2_ until 80–90% confluence was reached.

Next, the cells were harvested by treatment with 0.25% trypsin-0.02% EDTA (Gibco Life Technologies, Carslabad, CA USA) and seeded in 96-well plates (1 × 10^4^ cells per well) for MTT assay or in 6-well plates (4 × 10^4^ cells per well) for Trypan blue exclusion method. First the cells were pre-incubated for 24 h with 20 µM LA (physiologically relevant blood plasma concentration) then the cells were treated with serial pharmacological concentrations of ART (1, 2, and 8 µM) or DHA (1, 2, and 8 µM) with or without addition of two different LA concentrations (20 and 40 µM). The cells treated only with 20 µM LA were used as a control. All experimental settings were repeated with holo-transferrin (TRFi) at pharmacological concentration (50 µM). All cells were cultured for a period of 72 h.

### 4.4. MTT Assay

The cytotoxic effects of tested compounds were assessed by MTT (3-(4,5-dimethylthiazol-2-yl)-2,5-diphenyltetrazolium bromide) assay. This assay is based on the metabolic reduction of soluble MTT into an insoluble colored formazan product by mitochondrial dehydrogenase activity of viable cells. After 72 h incubation in the presence of experimental compounds the cells were incubated at 37 °C with MTT solution (0.5 mg/mL) for 4h. Obtained purple formazan product was dissolved in DMSO with isopropanol (1:1) and the optical density was measured at 570 nm. Each experiment was performed in triplicate. The cytotoxicity of compounds was presented as a percent of MTT reduced in treated cells vs. control cells. The relative MTT level (%) was calculated according formula [A]/[B] × 100. [A] express the absorbance of the test sample whereas [B] the absorbance of the control sample (untreated cells. IC_50_ values were calculated using CompuSyn version 1.0.

### 4.5. Trypan Blue Exclusion Assay

After a 72 h incubation with serial concentration of experimental compounds the cells were washed in PBS and harvested with trypsin. The viability was estimated by trypan blue exclusion assay using an automated cell counter (Countess Invitrogen). Experiments were performed in triplicate.

### 4.6. FITC Annexin V Binding Assay

The cells were cultured and harvested under the conditions mentioned in Cell Culture and Treatment and seeded in 6-well plates (2 × 10^5^ cells per well). After 24 h pre-incubation with 20 µM, LA cells were treated with tested compounds at IC_50_ concentrations of 72 h. The apoptotic effect of compounds was measured using the Annexin V:FITC assay kit (Sigma Aldrich) in accordance with the protocols provided. After 72 h treatment both floating and adherent cells were harvested. The floating cells were collected by centrifugation at 700× *g* for 5 min at 4 °C. Adherent cells were first trypsinized and then collected by centrifugation at 700× *g* for 5 min at 4 °C. Both fractions were resuspended in Annexin V binding buffer, pooled and incubated with FITC Annexin V and PI for 15 min at room temperature in the dark and analyzed by flow cytometry (Becton Dickinson). A total of 10,000 events were collected. Annexin V:FITC positive and PI negative cells were recognized as early apoptosis and Annexin V:FITC and PI positive cells as late apoptosis or necrosis.

### 4.7. Interleukin-6 Assay

The level of interleukin-6 (IL-6) secreted by SW480 and SW620 cells was measured by commercial human IL-6 ELISA kits (Diaclon SAS, Besancon Cedex, France). The cells were treated with IC_50_ concentrations of the tested compounds for 72 h. The untreated cells were used as the control. The IL-6 level in a cell culture supernatant was determined by an enzyme-linked immunosorbent assay, according to the manufacturer’s protocol. The experiment was repeated three times.

### 4.8. Statistical Analysis

The statistical calculations were performed by the means of Statistica 13.0 (StatSoft, Inc., Tulsa, OK, USA) program. Student’s *t*-test was used for quantitative comparison between studied groups. IC_50_ values were estimated by CompuSyn version 1.0. Results obtained from three separate experiments are presented as means ± SD, and considered statistically significant at *p* < 0.05.

## 5. Conclusions

To the best of our knowledge, this study is pioneer research carried out under conditions mimicking those occurring in cancer tissues in vivo and is the first study using LA as inherent cell culture component. It reveals equally high antitumor ART and DHA effects, which is remarkably underestimated in experiments performed at standard cell culture conditions, i.e., in the absence of LA and TRFi in the culture medium. It clearly demonstrates the need for obligatory presence of LA and TRFi in the culture media in all in vitro ferroptosis studies.

Our findings clearly indicate the high therapeutic potential of both studied compounds which may be used as adjuvant therapy in primary and metastatic colon cancer. Most importantly, contrary to classical cytostatics, this therapeutic management is not toxic for normal cells.

## Figures and Tables

**Table 1:** Cytotoxic activity (IC_50_, µM) of studied compounds estimated by the MTT assay ^a^.

Compound	Cancer Cells				Normal Cells
	SW480 ^d^		SW620 ^e^		HaCaT ^f^
	IC_50_^b^	SI ^c^	IC_50_	SI	IC_50_
ART	39 ± 2.75	5.2	42.8 ± 4.6	4.7	203 ± 16.2
ART-LA 20	17.5 ± 0.4	10.9	21.7 ± 7.3	8.8	191 ± 2.83
ART-LA 40	8.9 ± 3.1	16.4	18.4 ± 2.6	7.9	146.5 ± 6.38
ART-TRFi	4.58 ± 0.2	40.8	16.3 ± 6.1	11.6	187.1 ± 5.56
ART-LA 20-TRFi	2.36 ± 0.1	82.6	7.4 ± 0.11	26.1	195 ± 7.2
ART-LA 40-TRFi	0.69 ± 0.01	202	0.6 ± 0.04	225	142 ± 3.5
DHA	11.4 ± 1.67	10.1	11.9 ± 2.3	9.8	116 ± 5.5
DHA-LA 20	8.6 ± 0.9	16.1	9.8 ± 1.2	14.1	138 ± 3.1
DHA-LA 40	3.6 ± 0.3	13.8	1.27 ± 0.25	39.8	50.4 ± 3.4
DHA-TRFi	1.64 ± 0.07	78.7	10.3 ± 6.2	12.4	129.2 ± 0.5
DHA-LA 20-TRFi	0.38 ± 0.09	273	8.4 ± 0.9	12.3	104.4 ± 9.8
DHA-LA 40-TRFi	0.24 ± 0.02	288	0.14 ± 0.01	480	72.2 ± 1.6
DOX ^g^	0.75 ± 0.1	0.38	0.26 ± 0.1	1.11	0.29 ± 0.1

^a^ Data are expressed as mean ± SD. ^b^ IC_50_ (µM)—the concentration of the compound that corresponds to a 50% growth inhibition of cell line (as compared to the control) after culturing the cells for 72 h with the studied compound. ^c^ The SI (selectivity index) was calculated using the formula: SI = IC_50_ for normal cell line/IC_50_ for cancer cell line. ^d^ Human primary colon cancer cell line (SW480), ^e^ human metastatic colon cancer cell line (SW620), ^f^ human immortal keratinocyte cell line from adult human skin (HaCaT), and ^g^ the selected reference compound commonly used in cancer treatment (doxorubicin).

## Data Availability

Data is contained within the article.

## Abbreviations

AA	Arachidonic acid
ART	Artemisinin
CRC	Colorectal cancer
DHA	Dihydroartemisinin
LA	Linoleic acid
PUFAs	Polyunsaturated fatty acids
ROS	Reactive oxygen species
TRFi	Holo-transferrin
